# Ingrown Toenail in Children and Conservative Treatment Methods: A Case Report

**DOI:** 10.1111/jocd.70319

**Published:** 2025-09-03

**Authors:** Michalina Jakubczak, Klaudia Mazurek, Dominika Wcisło‐Dziadecka

**Affiliations:** ^1^ Department of Practical Cosmetology and Skin Diagnostics, Faculty of Pharmaceutical Sciences in Sosnowiec Medical University of Silesia Katowice Poland; ^2^ Doctoral School of the Medical University of Silesia Katowice Poland; ^3^ Podiatry Clinic Michalina Jakubczak Tarnowskie Góry Poland

**Keywords:** conservative treatment, ingrown toenail in children, nail diseases

## Abstract

**Background:**

Ingrown toenails are a disease that often begins in childhood. It occurs as the penetration of the nail plate into the surrounding tissue as a result of various factors. Patients often report to various specialists, including podiatrists and dermatologists, at an advanced stage of this disease.

**Aims:**

The aim of the study is to present the effectiveness of conservative treatment methods in children with ingrown toenails.

**Methods:**

In this study, we presented the case of a 12‐year‐old boy with ingrown toenails and visible inflammation around the toenails of both feet. Taking into account Scholz's classification, the severity of the disease in the right foot was assessed as 3B, and in the left foot as 3A. Non‐surgical conservative methods were used, such as cutting the wedge of the nail apparatus, applying a tamponade and implementing orthonyxia methods, which gave a quick therapeutic effect and the disappearance of unpleasant pain symptoms.

**Results:**

Conservative treatment methods using orthonyxia are highly effective, even in advanced stages of ingrowing nail plate. Medical procedures using conservative treatment techniques allow for quick help for pediatric patients in a safe manner and with a low risk of complications.

**Conclusions:**

Ingrown toenails can occur at any age, often including childhood, and conservative treatment methods should be the first possible treatment option.

## 1 Introduction

Ingrown toenails are one of many diseases related to the nail apparatus that may occur in childhood. Estimated data indicate that this problem affects on average 15%–20% of the world's population [1, 2, 3]. It involves the penetration of the nail plate into the surrounding soft tissues, causing inflammation. The discomfort felt by the patient may alternately subside and intensify as the disease develops and progresses [1, 2, 3, 4, 5, 6, 7, 8, 9]. To assess the severity of the disease, the Scholz classification is used (Table 1) [1, 4].

**TABLE 1:** Classification of ingrown toenails according to Scholz [1, 4].

Stage		Description
I	A unilateral condition	No inflammatory symptoms and little or no pain
	B bilateral condition	
II	A unilateral condition	Pain, inflammation, redness, swelling and tenderness of the nail folds and possible purulent discharge
	B bilateral condition	
III	A unilateral condition	Purulent inflammation and severe pain
	B bilateral condition	
IV	A unilateral condition	Paronychia, hypergranulation and severe pain
	B bilateral condition	
V	A unilateral condition	A postoperative condition in which the symptoms of ingrown toenail recur. Significant pain
	B bilateral condition	

Table 1 presents one of the available and used classifications of ingrown toenails, which allows both the assessment of the current severity of ingrown toenails and the progress of therapy. The clinical picture of an ingrown toenail may vary, depending on the patient's health condition and the advancement of the disease. The etiological factors that have a direct impact on the development of the disease include, among others: genetic predispositions, overweight or obesity, adolescence, mechanical injuries within the nail apparatus, use of inappropriate footwear, improper shortening of nails, congenital and acquired foot deformities, overload conditions, hyperhidrosis, nodular subungual lesions, metabolic and circulatory system diseases, medications used, and large hygiene neglect [2, 5].

Assessment of the advancement of the disease is the key issue in choosing a therapeutic method, as shown in Table 2.

**TABLE 2:** Therapeutic options depending on the stage of ingrown toenail in relation to the classification according to Scholz [1, 7].

Stage of advancement	Therapeutic options	
	Conservative treatment	Surgical treatment
1A 1B	+	−
2A 2B	+	−
3A 3B	+	+
4A 4B	+	+
5A 5B	+	+

Ingrown toenail is a multi‐stage condition that may develop and intensify as a result of one or more etiological factors at any stage of the patient's life, including childhood. The multitude of factors predisposing to the occurrence of the disease, as well as the potential risk of recurrence of nail plate dysfunction, determines the selection of the appropriate form of treatment [3, 6].

The generally accepted forms of treatment for ingrown toenails include conservative and surgical methods. The main surgical methods include:

- extraction (total resection)—detachment of the nail plate from the matrix and nail bed,
- wedge resection—wedge‐shaped excision of the lateral fragments of the nail plate along with part of the nail folds and, if necessary, the nail bed,
- Zadik procedure—complete excision of the nail matrix, leading to the absence of the nail plate,
- marginal resection—excision of the lateral part of the nail plate, most often combined with chemical or physical ablation of part of the matrix and/or part of the nail bed,
- amputation of the phalanx—amputation of the distal part of the phalange,
- plastic surgery of the periungual folds—removal of excess tissue from the periungual folds, leaving the nail bed intact,

as well as independent physical methods (laser, radiofrequency, freezing, electrocoagulation) and chemical methods (phenol, 5 or 10% sodium hydroxide, trichloroacetic acid, nitrogen peroxide or silver nitrate) [1, 3, 6, 7].

The available methods of conservative treatment of ingrown toenails include:

- tamponade—placed between the nail plate and the periungual folds of the dressing material, for example, sterile gauze compresses, non‐woven compresses, fleece tape, ligasano,
- permanent tamponade/tubing/splinting of the lateral edge of the nail plate—placing solid materials, for example, sulci protector tube, additionally protected with a light‐curing material, between the nail plate and the periungual folds,
- taping of periungual folds—pulling the nail folds downwards using kinesiotaping tapes,
- threading the nail plate—lifting the distal parts of the nail plate using dental floss and glued dressings,
- acrylicizing of the nail plate—protection of the nail plate with its side edges using medical acrylic, in a raised position,
- use of local agents in the form of dressings, disinfectants, or products (ointments) accelerating healing,
- shoe replacement,
- side cutting of the nail plate—cutting the side part of the nail plate, which is an irritating element in the periungual fold; the decision to perform this type of procedure depends on the advancement of the disease and the ability to perform this type of activity,

orthonyxia—is a non‐invasive method for correcting transverse curvature of nail plates [1, 3, 4, 6].

Ortonyxia is a set of methods based on the use of orthonyxic clasps on the nail after the involved part of the nail has been excised. These are temporary metal and/or non‐metal implants made of various materials, applied to the nail plate or attached under the nail plate, which are intended to correct the shape of the nail plate and slightly lift it. Orthonyxia has a minimal impact on patient's quality of life and they can participate in most physical activities right after the treatment. In addition, the use of this method does not require local anesthesia, and after the procedure the patient feels almost immediate reduction in pain [10, 11].

## 2 Materials and Methods

A 12‐year‐old boy and his parents came to the podiatry office with visible inflammation around the toenails of both his feet. The patient was under the care of a pediatrician. Local antibiotic therapy was used for the last 6 months, and then a podiatry consultation was recommended. Family history is negative for ingrown toenails, the boy's body weight is normal, there is no family history of obesity. The etiological factors of the disease were determined. In this case, they were: improper shortening of nails by the child, too tight shoes (which, however, had been worn for a relatively short time before the changes in the nails occurred), as well as episodic states of hyperhidrosis of the feet. Moreover, the boy was entering puberty and thus a time of more intense growth.

**FIGURE 1:**
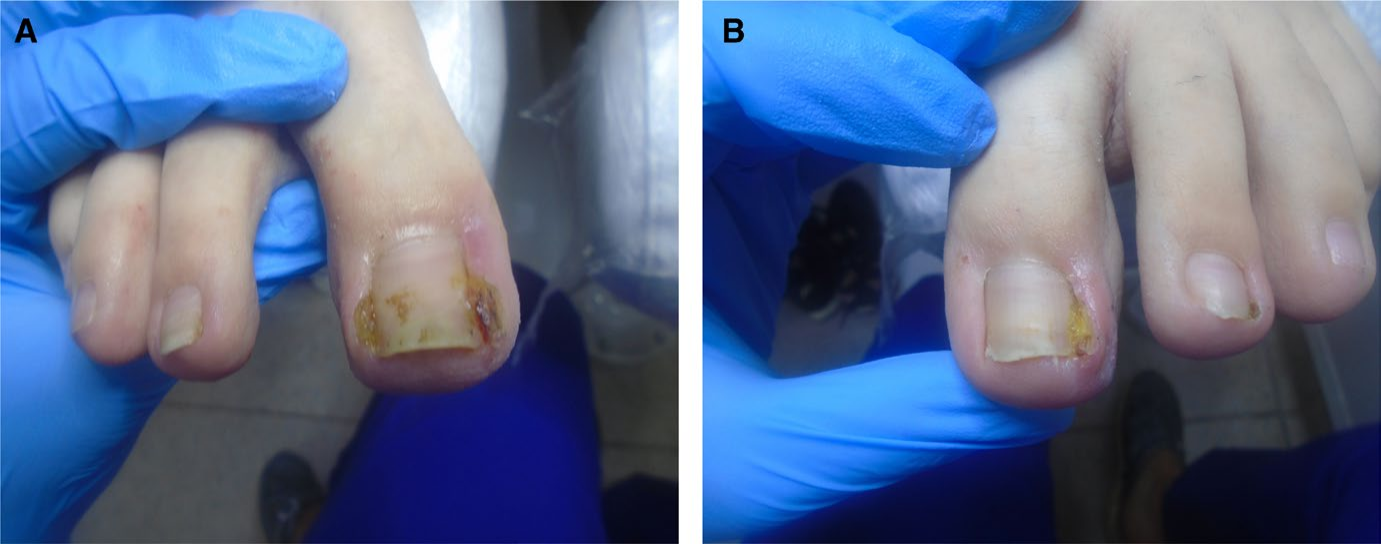
(A) The condition of the patient's right big toe on the day of the first visit. Ingrown toenail stage 3B according to Scholz classification (B) The condition of the patient's left big toe nail on the day of the first visit. Ingrown toenail stage 3A according to Scholz classification.

Taking into account Scholz's classification, the severity of the disease in the right foot was assessed as 3B, and in the left foot as 3A, as shown in Figure 1A,B, respectively.

**FIGURE 2:**
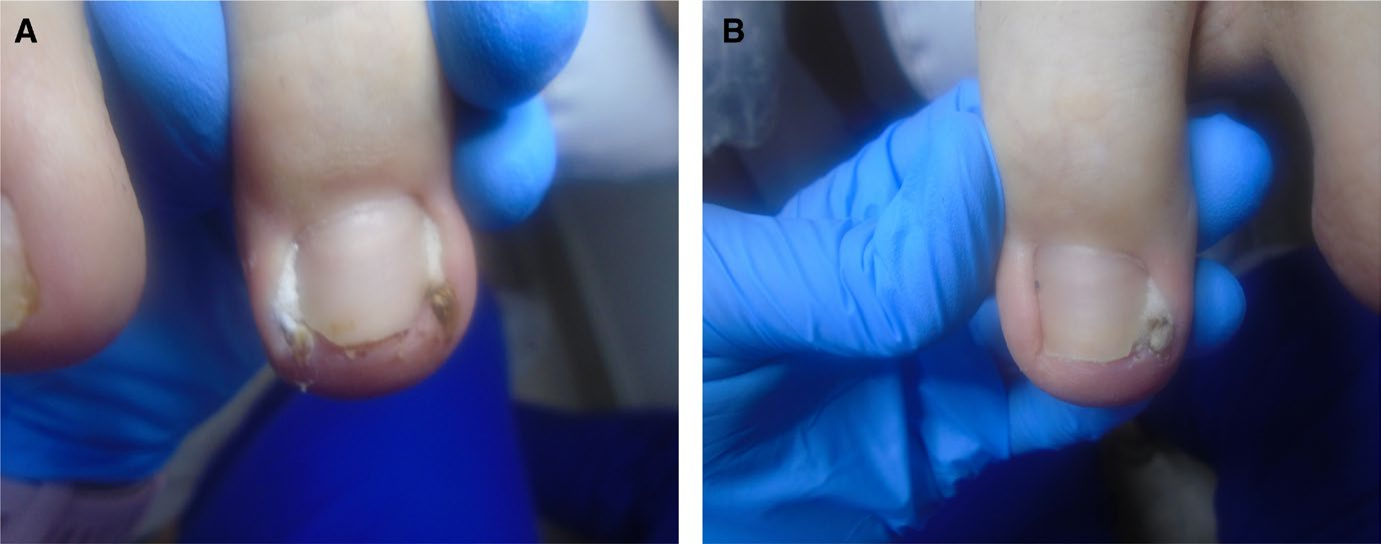
(A) Condition after the first procedure of shortening and wedge cutting of the ingrowing nail on both sides of the right foot. The photo also shows the tamponade in place. (B) Condition after the first treatment of shortening and wedge cutting of an ingrown toenail on one side of the left foot. The photo also shows the tamponade in place.

During the first visit, a podiatry treatment was performed, including full cleaning of the large nail area using sterile tools. A unilateral and bilateral wedge trim was also performed—depending on the condition of the nail apparatus. In addition, the periungual folds were protected with tamponade—a form of conservative treatment. The procedure did not require local anesthesia. The boy felt a reduction in pain almost immediately after the procedure. Additionally, recommendations were issued regarding the frequency of disinfection of the nail area (disinfectant preparation containing octenidine and phenoxyethanol, application: two to three times a day), instructions were provided on to replace the tamponade on your own, and local dressings with silver ions were introduced to supply the external nail apparatus. In addition, the patient was not advised to take any oral medications, including painkillers, anti‐inflammatory drugs or antibiotics. The patient's condition immediately after the first podiatry treatment is presented in Figure 2A,B.

The first follow‐up visit took place after 12 days. A significant reduction in inflammation was observed and further use of disinfectants and tamponade was recommended, without the necessary external occlusion of the big toes of both feet.

After the inflammatory symptoms had fully subsided (after 5 weeks), it was decided, in consultation with the patient's parents and with their consent, to implement an additional method of conservative treatment in the form of orthonyxia.

The direct goal of the treatment using the brace was to achieve a change in the shape of the nail plate by lifting and flattening it, eliminating the effect of ingrown nails. A special advantage of treatment with this method is the permanent relief of pressure caused by the side edges of the nail plate on the periungual folds during therapy. Thanks to this, the patient feels relief and no discomfort during everyday functioning already in the first days after the application of the metal implant on the surface of the nail plate. The application of the brace was carried out without any complications or discomfort for the patient. The first period of wearing the implant had a positive effect on the patient's well‐being and thus on the satisfaction of the parents. The initial schedule of visits included meetings at least once a month, with the exception of emergency situations, for example, sudden mechanical damage to the nails undergoing therapy or damage to the brace. In this case, there were no emergency situations during the therapy.

**FIGURE 3:**
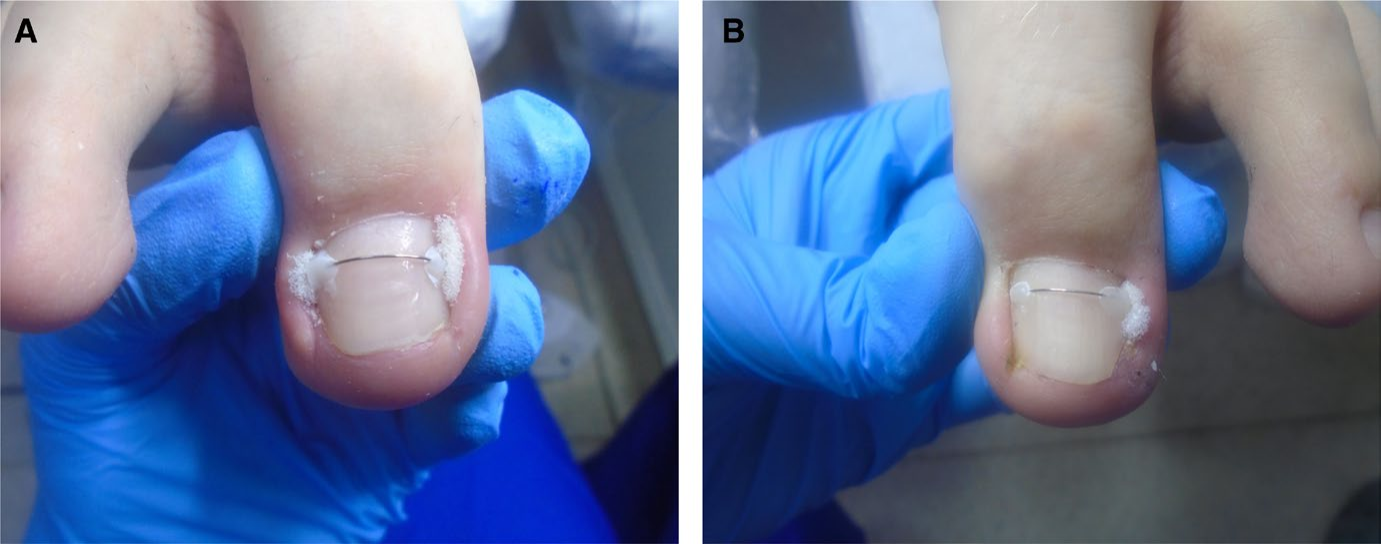
(A) Condition of the nail apparatus during conservative treatment using orthonyxia with tamponade. Condition after applying 1 brace to the nail of the big toe of the right foot. (B) Condition of the nail apparatus during conservative treatment using orthonyxia with tamponade. Condition after applying 1 brace to the nail of the big toe of the left foot.

**FIGURE 4:**
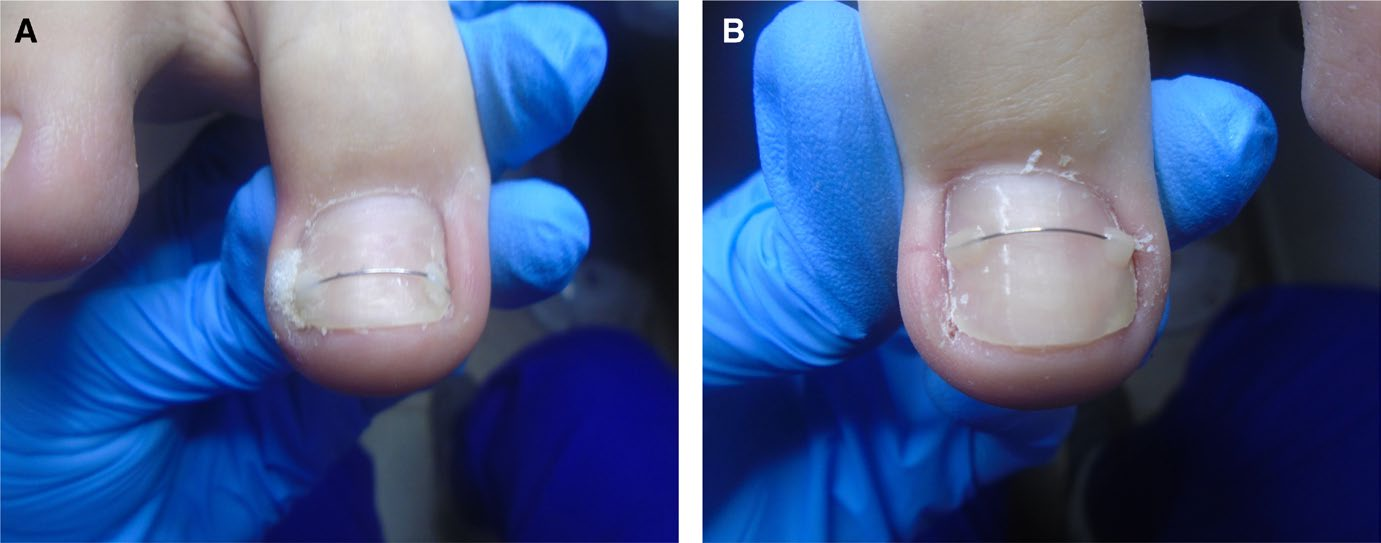
(A) Patient undergoing conservative treatment using orthonyxia, after 2 months. Tamponade visible on one side. (B) Patient undergoing conservative treatment using orthonyxia, after 2 months. Left foot.

The subsequent effects of the treatment are presented in Figures 3A,B, 4A,B.

In the attached graphics, you can see a significant improvement in the appearance of the nail bed. It is also important that the lack of discomfort associated with everyday functioning translates positively into the progress of therapy and satisfaction with the chosen method of treating ingrown toenails. Already at this stage of treatment, you can see that the shape of the nail and the nail folds have changed dramatically in appearance, compared to the first visit.

Orthonyxia methods are characterized by long‐term effects. This means that the longer the implant is on the nail plate, the greater the effects of nail plate shape correction are achieved. The low invasiveness of the treatment and the lack of discomfort mean that conservative treatment does not require special caution from the patient during the therapy, and the therapy itself progresses.

Orthonyxia, like any other therapeutic method, is potentially burdened with the risk of side effects such as onycholysis, unsightly change in nail shape and possible escalation of nail damage during mechanical trauma, or possible scratches/wounds associated with displacement or separation of the metal implant from the nail plate. Adverse reactions are very rare in this treatment method. Their occurrence is largely a result of the correct performance of the procedure and compliance with post‐treatment recommendations. In the case of our 12‐year‐old patient, no adverse effects occurred, thanks to which the therapy could be continued.

**FIGURE 5:**
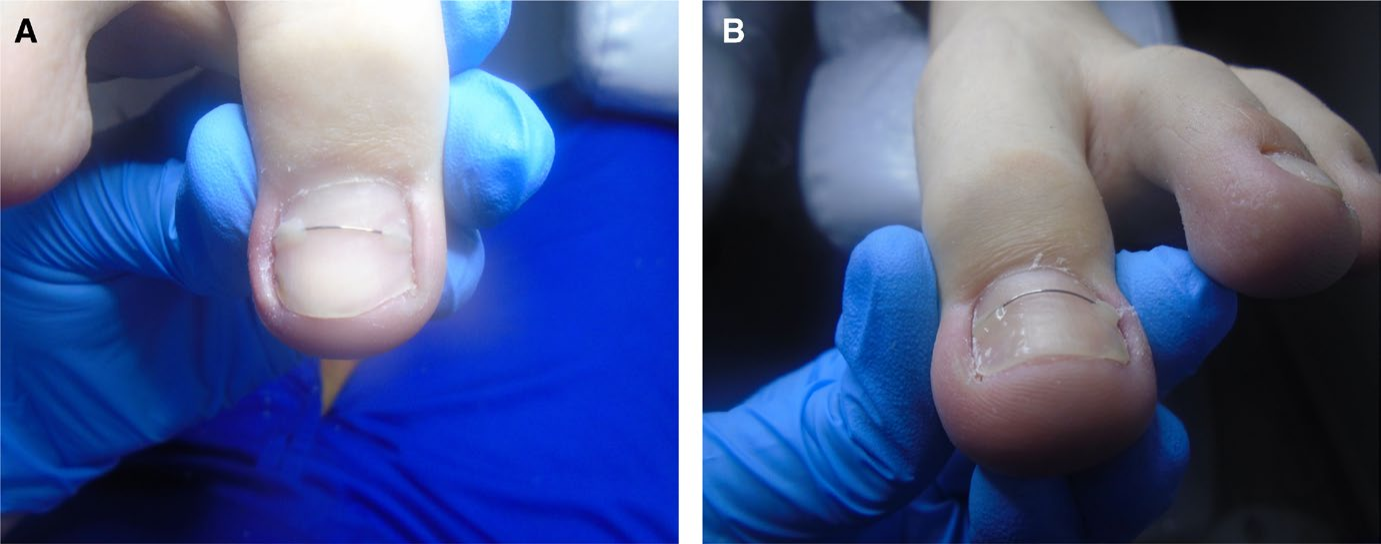
(A) The condition of the big toe of the right foot 4 months after starting treatment with conservative methods and 3 months after starting orthonyxia. No visible or felt symptoms of ingrowing nail plate. (B) The condition of the big toe of the left foot 4 months after starting treatment with conservative methods and 3 months after starting orthonyxia. No visible or felt symptoms of ingrowing nail plate.

The use of both conservative treatment methods had a positive effect on the condition of the nail apparatus, as shown in the figures below (Figure 5A,B).

The last photos show the achieved therapeutic effect, described by the patient as satisfactory. The large change in the shape of the nail plate means that the possibility of recurrence of inflammation is significantly minimized. It is worth noting the big difference in the appearance of the entire nail apparatus, compared to the photos from the first visit.

After completing the therapy, the patient and his parents emphasized that they were satisfied with the choice of the treatment method and the effects achieved by it. The patient's well‐being improved already in the first month of conservative treatment in the form of orthonyxia methods, which had a positive impact on the therapeutic possibilities.

## 3 Discussion

The problem of ingrown toenails can occur in patients of all ages, but research shows that it is particularly common in young people aged 11–30 [9]. Therefore, it seems reasonable to select therapeutic methods of reducing this problem that are both effective and associated with the shortest possible convalescence. Many publications are devoted to the effectiveness of surgical methods in the treatment of ingrown toenails. Marginal or complete resection of the nail plate with phenolization of the matrix is particularly popular. However, children, pregnant women, or patients with severe systemic diseases cannot be subjected to this method of treatment [9]. Therefore, the choice of conservative methods in these groups of patients seems to be the best therapeutic option.

The use of conservative treatment in the case of ingrown toenails is still a less popular form of treatment among all available options. For children and adolescents, this is one of the safest and most effective solutions. The presented case confirms the positive impact of the applied conservative treatment methods on the nail apparatus. A positive assessment of the podiatry methods, such as orthonyxia and tamponade, presented in this work can also be found in other publications. The studies by Kuros et al. show high effectiveness in treating ingrown toenails in adolescents using orthonyxia methods and correspond with the results of our research [9]. Similar results are also presented by Jakubowska et al., who prove that the less invasive nature of the procedures is used primarily in patients with chronic diseases, in adolescents, children, and in the elderly, in whom the use of the surgical method is associated with too high of a risk of permanent damage to the nail apparatus or the occurrence of complications or permanent aesthetic defects [4]. Rosien et al. also draw attention to the safety and effectiveness of conservative methods in the treatment of ingrown toenails in special patients, namely in people with wound healing problems [12].

According to Thakur et al., there is no ideal method of treating ingrown toenails that would be 100% effective as a surgical or conservative treatment method [6]. The presented case proves that the combination of several non‐surgical methods brings a quick therapeutic effect and thus causes a relatively quick disappearance of unpleasant symptoms, which is particularly important in relation to children and adolescents. It is necessary to mention the undoubted advantages of treatment using the orthonyxia technique, which include the lack of obstacles in everyday functioning, protection against the recurrence of inflammatory complaints, little involvement in the therapy on the part of the patient, or no need to give up daily activities, such as exercise. The advantages mentioned above are of particular importance for patients who are adolescents or children.

Research by Kasuya et al. showed that surgical treatment is characterized by a lower risk of recurrence compared to conservative treatment [7]. However, it is an invasive procedure that involves a longer recovery time, and the risk of recurrence cannot be completely eliminated. In addition, it is associated with pain, a long healing period, and the risk of bone infection. Conservative methods are much less invasive, provide quick pain relief, have a low risk of post‐operative complications, and ensure full functionality. In turn, studies conducted by Liu and Huang prove the effectiveness of treatment with conservative methods using orthonyxia for ingrown toenails [8]. They prove the validity of implementing these forms of treatment first.

In the case of young patients (children and teenagers), ingrown toenails may be episodic, often caused by external factors, the elimination of which significantly reduces the risk of recurrence of the disease. The use of conservative treatment methods in the first place in young people may turn out to be one of the safest and longest‐lasting solutions.

As Rampal et al. point out, many developmental and physiological defects in the feet of children carry a greater risk of developing ingrown toenails [13]. In such a case, it will be necessary to provide children with physiotherapeutic and orthopedic care, and from a podiatry perspective, the optimal choice will be conservative treatment, the pace of which can be individually adjusted to the changing foot motor skills. Márquez‐Reina et al. reached interesting conclusions by combining surgical methods with subsequent conservative treatment. The results of their research indicate that such a therapeutic plan increases the effectiveness of both methods and ensures long‐lasting effects in patients [14]. They also showed that chemical ablation alone is less effective without the implementation of orthonyxia. This is another thesis supporting the use of non‐surgical methods for pediatric patients.

## 4 Conclusions

Ingrown toenails can occur at any age, often including childhood, and conservative treatment methods should be the first possible treatment option.

Conservative treatment methods using orthonyxia are highly effective, even in advanced stages of ingrowing nail plate. They allow for quick help for pediatric patients in a safe manner and with a low risk of complications.

An important element of the treatment of ingrowing nail plate is the correction of its shape, and this is possible thanks to the use of orthonyxia methods.

Despite the long duration of the treatment, it offers satisfying effects and no discomfort in functioning during the therapy, and also requires little patient involvement, which makes this treatment option a good choice for the patient.

## Author Contributions

All authors were responsible for the concept and design of the study, collection and collation of data, analysis and interpretation of data, write an article, reviewing this article and graphics performance.

## Ethics Statement

The authors confirm that the ethical policies of the journal, as noted on the journal's author guidelines page, have been adhered to and the appropriate ethical review committee approval has been received. The research project was approved by the Ethics Committee of the Medical University of Silesia in Katowice (Resolution no. BNW/NWN/0052/KB1/60/II/22/23, dated 18.07.2023).

## Consent

The patient and the patient's parents consented to participate in the study in a statement attached to his medical records.

## Conflicts of Interest

The authors declare no conflicts of interest.

## Data Availability

The data that support the findings of this study are available from the corresponding author upon reasonable request.
